# Diagnostic accuracy of tumor necrosis factor-alpha, interferon-gamma, interlukine-10 and adenosine deaminase 2 in differential diagnosis between tuberculous pleural effusion and malignant pleural effusion

**DOI:** 10.1186/1749-8090-9-118

**Published:** 2014-07-01

**Authors:** Mingying Li, Helin Wang, Xia Wang, Jian Huang, Junxiang Wang, Xiue Xi

**Affiliations:** 1 The First Affiliated Hospital of Xinxiang Medical University, No.88 Jiankang Road, Weihui 453100, Henan Province, China

**Keywords:** Tuberculous pleural effusion, Malignant pleural effusion, Differential diagnostic significance, Tumor necrosis factor-alpha, Interferon-gamma, Interlukine-10, Adenosine deaminase 2

## Abstract

**Background:**

The current study was performed to investigate the potential biomarkers for the differential diagnosis of tuberculous pleural effusion (TPE) and malignant pleural effusions (MPE).

**Methods:**

Among ninety patients (n = 90) involved in the study, 47 with tuberculous pleural effusion aged from 18 to 70 and 43 with secondary malignant pleural effusion aged from 34 to 78. We tested the pleural levels of TNF-α, IFN-γ and IL-10 as well as the enzyme activity of ADA_2_, and then we compared the differential diagnostic efficiencies of those biochemical parameters with ADA between the two groups.

**Results:**

Our results show that, the concentrations of pleural TNF-α (45.55 ± 15.85 ng/L), IFN-γ (114.97 ± 27.85 ng/L) as well as activities of ADA_2_ (35.71 ± 10.00 U/L) and ADA (39.39 ± 10.60 U/L) in tuberculous group were significantly higher compared to malignant group. Furthermore, according to the ROC curve analysis the thresholds of TNF-α, IFN-γ, ADA_2_ and ADA were found to be 30.3 ng/L, 103.65 ng/L, 29.45 U/L, and 39.00 U/L, respectively. TNF-α, IFN-γ and ADA_2_ yielded better sensitivity, specificity, and accuracy of the diagnosis than ADA. Our investigation further revealed that the combinations of TNF-α and ADA_2_ further increased the specificity and accuracy for the differential diagnosis.

**Conclusion:**

In conclusion, we found that TNF-α, IFN-γ, ADA and ADA_2_ all increased in TPE. Combinations of the TNF-α and ADA_2_ yielded the best specificity and accuracy for the differential diagnosis of TPE from MPE. Our investigation suggests that the applications of TNF-α together with ADA_2_ may contribute to more efficient diagnosis strategies in the management of discrimination between tuberculous and malignant pleural effusions.

## Background

Most of pulmonary and systemic diseases may be associated with pleural effusion. It is a common clinical problem and it has been estimated that there are >800,000 cases/year in the USA [1]. Tuberculosis and malignant diseases involving the pleura are the leading causes of pleural effusion, which has an occurrence of 49.6% and 29.6%, respectively, especially in the under developed areas among all pleural effusion cases, respectively [2-5]. Thus, it is of great clinical significance to explore efficient biochemical markers for making differential diagnosing of tuberculous pleural effusion (TPE) from malignant pleural effusion (MPE). The diagnosis of TPE is made by detecting *Mycobacterium tuberculosis* in the pleural fluids and/or pleural biopsy specimens, or demonstrating caseation granulomas in pleura [6]. However, only 10-35% of biological culture and 20-81% of molecular tests reveal mycobacteria in pleural fluids, and pleural biopsy demonstrates granulomas in 56-82% of samples [7-10]. In addition, the financial problem is a burden for the patients as well. Furthermore, the discrimination from MPE, which is mainly diagnosed based on the pathological methods, is still a challenge.

It is reported that adenosine deaminase (ADA), tumor necrosis factor-alpha (TNF-α), interferon-gamma (IFN-γ), interlukine-12 (IL-12), interlukine-18 (IL-18), interlukine-10 (IL-10), interlukine-27 (IL-27), Immunosuppressive acidic protein (IAP), and soluble IL-2 receptor could serve as differential diagnosis biomarkers for pleural effusion caused by TB or malignant diseases [7,11,12]. Adenosine deaminase (ADA), a purine-degrading enzyme implicated in mononuclear phagocyte maturation, has been reported to accumulate in the pleural fluid of TB patients and being predict TB pleurisy with high sensitivity and specificity at 95% and 90% respectively [6]. The accumulation of ADA in pleural fluid results mainly from one of its isoforms, ADA_2_, with which a diagnosis of tuberculous pleurisy could be verified [13]. In the past decade, researchers demonstrated that both tuberculous and malignant pleural effusions show marked increase of TNF-α [14-17]. And the up-regulated IFN-γ and IL-10 in fluid can be diagnostic parameters for tuberculous pleural effusion as well. Most recently, interlukine (IL)-27, a member of IL-12 family, has been verified useful in diagnosing TPE or discriminating pleural effusions caused by tuberculosis from other medical situations [12,18]. However, none of those is widely used in clinical practice currently but it is only restricted to research settings.

In this present study, we aimed at exploring the potential series of diagnostic biomarkers. In order to figure out the clinical significance of these diagnostic parameters for the discriminating diagnosis of tuberculous and malignant pleural effusions, concentrations of TNF-α, IFN-γ and IL-10 and enzyme activity of ADA_2_ were measured and compared with ADA activity.

## Methods

### Patients and sample collection

A total of 90 patients (n = 90) admitted in Henan Tuberculosis Hospital between Jun. 2010 and May. 2012 were involved in this research (Table 1). All patients have been diagnosed based on clinical symptoms, pleural effusion analysis, and/or pleural biopsy specimen observation. Accordingly, the subjects were determined as tuberculous pleural effusion based on the presence of either positive staining or culture for *M. tuberculosis* in the pleural fluid, sputum or pleural biopsy specimen or caseating granulomas on pleural biopsy. Secondary malignant pleural effusion diagnosis was based on the determination of malignant cells on cytological examination or in a biopsy specimen, or by histologically determined primary malignance with the exclusion of any other cause of pleural effusion. Among all the 43 patients, there were 26 patients with lung cancer (60.5%), 6 patients with breast cancer (14.0%), 7 metastatic cancer patients with unknown idiopathy (16.3%), and 4 patients with stomach, pancreatic or ovary cancer (9.3%). All pleural fluid samples were collected by thoracentesis prospectively before the patients undergone any medical treatments. Collected samples from all the patients were centrifuged and kept in freezer in −70°C.

**Table 1:** Clinical information of patients

**Group**	**Case**	**Gender**		**Age**
		**M**	**F**	**(**$\bar{x}$**±s)**
Tuberculous	47	29	18	51.3 ± 11.8
Malignant	43	23	20	55.5 ± 12.4

Comparison between gender *χ*^2^ = 0.263,*P* > 0.05.

Comparison between age *t =* 1.646*,P* > 0.05.

This study was approved by the Ethics Committee of Henan Tuberculosis Hospital. Study participants and/or their legal guardians granted written-informed consent.

### Determination of cytokines’ concentration and enzyme activities of ADA and ADA_2_

Enzyme-linked immunosorbant assay (ELISA) was performed according to manufacturer’s instructions to determine the pleural concentrations of TNF-α, IFN-γ and IL-10 using commercial kits (Biosource). Pleural enzyme activity of ADA and ADA_2_ were determined by spectrophotometric method according to Muraoka’s [19] method. In detail, the catalyzed enzyme activity of ADA or ADA_2_ was determined by quantifying hypoxanthine liberated from the substrate (1 U/L) under the atmosphere of 37°C and pH7.1. The substrate of ADA was composed of adenosine (6 mM), γ- oxoglutarate (1.1 mM), ADP (1 mM) and glutamate dehydrogenase (18 U/L). And the ADA_2_ activity was determined by the same substrate with the presence of erythro-9-(2-hydroxy-3- nonyl) adenine hydrochloride (EHNA, 0.1 mM). The enzyme activities absorbance was read at 340 nm using automatic biochemical analyzer (Olympus AU600). Here, the enzyme activity (1 U/L) was defined as 1 μM hypoxanthine liberating from catalyzed adenosine per minute. All samples were assayed in duplicate.

### Statistical analysis

Computerized statistical analyses were performed using the Statistical Package for the Social Sciences (SPSS), version 17.0. The variances of different biomarkers among different groups were analyzed using Mann–Whitney *U*-test, the comparison of sensitivity and specificity was analyzed by *χ*^2^ test. Sensitivity and specificity values providing the best test performance and the area under the curve (AUC) were calculated using a receiver operating characteristic (ROC) curve analysis. In all statistical analyses, a two-tailed *p* value ≤0.05 was considered statistically significant. Data were presented as mean ± standard error (SE).

## Results

### Clinical information of patients

In our study neither age nor gender has any statistical significance between groups (Table 1).

### Concentration of pleural cytokines and enzyme activities of ADA and ADA_2_

We assayed the concentrations of pleural TNF-α, IFN-γ, IL-10 as well as the activities of ADA and its isoenzyme, ADA_2_. Then we compared the diagnostic efficiencies of the cytokines and ADA_2_ with ADA, which is well known for the high diagnostic value for tuberculous pleural effusion. The level of TNF-α was up-regulated significantly in tuberculous group from 17.18 ± 4.84 ng/L to 45.55 ± 15.85 ng/L compared to malignant group. In addition, the pleural IFN-γ was higher in tuberculous group (114.97 ± 27.85 ng/L) than in malignant group (87.15 ± 18.77 ng/L) as well (p < 0.001). The enzyme activities of ADA in tuberculous and malignant pleural fluid were 39.39 ± 10.60 U/L and 32.16 ± 6.99 U/L respectively. The ADA_2_ activity was 35.71 ± 10.00 U/L and 20.87 ± 4.53 U/L in tuberculous and malignant pleural effusion respectively as shown in Table 2. However, there was no statistical significance on the level of pleural IL-10 between these two groups.

**Table 2:** **Pleural fluid enzyme activities of ADA and ADA**_**2**_**, concentrations of TNF-**α**, IFN-**γ **and IL-10 (±s)**

**Group**	**n**	**ADA (U/L)**	**ADA**_**2 **_**(U/L)**	**ADA**_**2**_**/ADA (%)**	**TNF-α (ng/L)**	**IFN-γ (ng/L)**	**IL-10 (ng/L)**
Tuberculous	47	39.39 ± 10.60	35.71 ± 10.00	90.63	45.55 ± 15.85	114.97 ± 27.85	6.12 ± 2.31
Malignant	43	32.16 ± 6.99	20.87 ± 4.53	64.89	17.18 ± 4.84	87.15 ± 18.77	6.20 ± 2.29
*t*		5.74	12.76	56.89	12.26	5.28	0.994
*P*		<0.001	<0.001	<0.001	<0.001	<0.001	>0.05

n = number of subject; Values are mean ± SD.

### Differential diagnostic values of TNF-α, IFN-γ, ADA and ADA_2_

**Figure 1:**
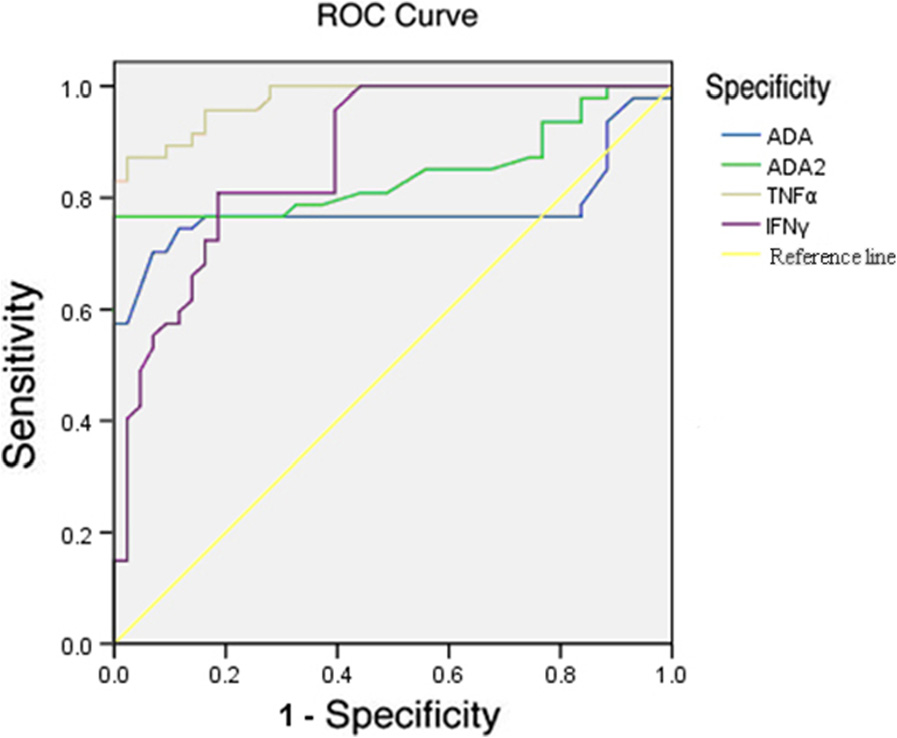
**Receiver Operative Characteristic (ROC) curves of pleural TNF-α, IFN-γ, ADA and ADA**_**2**_**.** The cut-off values of TNF-α,IFN-γ, ADA and ADA_2_ were determined by the best sum of sensitivity and specificity on the ROC curve. Diagonal line indicates the line of no discrimination.

ROC curve analysis was introduced to evaluate the cut-off values of pleural TNF-α, IFN-γ, ADA and ADA_2_ (Figure 1). The thresholds were found to be 30.3 ng/L, 103.65 ng/L, 39.00 U/L and 29.45 U/L respectively (Table 3). Unfortunately, when compared to ADA, no statistical significance was found on the area under the curve (AUC) value of TNF-α, IFN-γ and ADA_2_ (p > 0.05) (Table 3). But the AUC for all of these bio parameters are greater than 0.5, TNF-α (0.976), IFN-γ (0.831) and ADA_2_ (0.844), indicating that TNF-α, IFN-γ and ADA_2_ are reliable for the clinical diagnosis of TPE.

**Table 3:** **Differential diagnostic significance of TNF-**α,** IFN-**γ **and ADA**_**2**_

**Bio-parameter**	**AUC**	**Cut-off**	**Sensitivity (%)**	**Specificity (%)**	**Accuracy (%)**
TNF-α	0.976*	30.3 (ng/L)	83.0	97.0	90.0
IFN-γ	0.831^#^	103.65 (ng/L)	80.9	81.4	81.1
ADA_2_	0.844^##^	29.45 (U/L)	76.6	97.0	86.7
ADA	0.779	39.00 (U/L)	76.6	83.7	80.0

Compared with ADA*χ^2^ = 0.201, p > 0.05; ^#^*χ*^2^ = 0.655, *p* > 0.05; ^##^*χ*^2^ = 0.81, *p* > 0.05.

With a cut-off value of 30.3 ng/L, the sensitivity of TNF-α for differential diagnosis of TPE from MPE was 83%, with the specificity of 97% and accuracy of 90% (Table 3). Also as shown in Table 3, with a cut-off value of 103.65 ng/L, the sensitivity of IFN-γ was 80.9%, specificity was 81.4% and accuracy was 81.1%. Furthermore, our data indicated that ADA_2_ yielded slightly better specificity of 97% and accuracy of 86.7% than ADA but equivalent sensitivity of 76.6% for the differential diagnosis of the diseases (Table 3).

### Combined-diagnostic value of TNF-α, IFN-γ and ADA_2_

The question of the possibility of the combinations of two or three of the parameters would improve the diagnostic sufficiency for differentiating tuberculous pleural effusion from malignancy is raised. As a result, we further investigated the potential combined-diagnostic value of TNF-α, IFN-γ and ADA_2._ Parallel tests were introduced in our analysis to evaluate the differential diagnostic significances of the biochemical parameters of interests for tuberculous or malignant pleural effusion. When two or three parameters of TNF-α, IFN-γ and ADA_2_ were used for diagnosis, the measurement of TNF-α plus ADA_2_ yielded the highest specificity (97.7%) and accuracy (88.3%) but slight increase in sensitivity (78.9%) (Table 4) for discriminating tuberculous pleural effusion from malignant pleural effusion.

**Table 4:** Combined-biochemical parameters’ diagnostic value analysis

**Bio-parameter**	**Sensitivity (%)**	**Specificity (%)**	**Accuracy (%)**
TNF-α + IFN-γ	81.9	89.5	85.6
TNF-α + ADA_2_	78.9	97.7	88.3
IFN-γ + ADA_2_	78.7	89.5	83.9
TNF-α + IFN-γ + ADA_2_	80.0	92.2	85.9

## Discussion

Tuberculosis and secondary malignant diseases are the leading causes of pleural effusion. Although the pleural fluids were mainly composed of lymphocytes both in TPE and MPE, clinical treatments and prognosis vary significantly. Thus, differential determination plays a key role in the clinical procedure of pleural effusion. Tuberculous pleurisy is diagnosed based on the positive culture of pleural *M. tuberculosis* or demonstrating the characteristic caseation granulomas in pleural biopsy specimens [6]. However, histological or bacteriological confirmation for tuberculous pleurisy is of lower sensitivity or accuracy. The lack of more efficient diagnostic criteria drove investigators to explore a specific marker for diagnosis of diseases.

In the past decades, series of bio-parameters were reported to play a role in the differential diagnosis of tuberculous and malignant pleural effusions. Among which, ADA, a purine-degrading enzyme that catalyze the conversion of adenosine and deoxyadenosine to inosine and deoxyinosine with the release of ammonia, is the most sensitive and specific marker for tuberculosis pleurisy diagnosis [20-23]. Meanwhile, ADA_2_, isoform of ADA, was reported to be a useful biochemical marker for the early diagnosis of tuberculous pleural effusion and differentiate tuberculous pleural effusion from non-tuberculous origins efficiently [24]. As shown in this study, the sensitivity and specificity yielded for ADA on the diagnosis of tuberculosis pleurisy are 76.6% and 83.7%, respectively, which are in agreement with previous studies. Based on these results, we further confirmed the clinical significance of ADA_2_. Meanwhile, the ratio of ADA_2_ to the total ADA (mean: 90.63%) increased significantly in the tuberculosis group, indicating the increased pleural ADA_2_ accounts for the high activity of ADA in the effusion fluid. As a delayed hypersensitivity reaction, TB was resistant by Th1 type immune response of the body, which enables ADA_2_ up regulated in the pleural fluid. As shown in our study, pleural ADA_2_ yielded the great sensitivity of 76.6% and the highest specificity of 97% on the diagnosis of tuberculous pleurisy. Unfortunately, no statistical significance was found when compared with ADA. However, it was reported that ADA activity may be lower at the early stage of tuberculous pleurisy and the high activities may decrease long after onset of the disease as well [13]. Recently it was reported that the increased ADA activity was not restricted in tuberculous pleurisy but in the pleural fluid associated with pyothorax, lymphoma or ILS [6]. Therefore we further investigated other diagnostic bio parameters, hoping to discover a reliable biomarker for the diagnosis of tuberculosis associated pleural effusion.

Tumor necrosis factor-α (TNF-α), a small polypeptide, functions on biological and immunological processes. It is a pro-inflammatory cytokine, which is synthesized by lymphocytes and monocytes/macrophages [25]. As a Th1 subset member, it plays an important role in anti-inflammation reaction and tumor resistant process. Previous literatures demonstrated significant accumulated TNF-α in pleural fluid of TB [26] comparing to the malignant pleural effusion. As expected, we detected the up-regulated pleural level of TNF-α (45.55 ± 15.85 ng/L) in the TB pleural fluid, which is significantly higher than that of malignant (17.18 ± 4.84 ng/L) group. Meanwhile, with a cut off of 30.3(ng/L), the sensitivity and specificity of TNF-α for tuberculous pleurisy diagnosis were determined to be the highest, 83% and 97% respectively, which is in agreement of previous findings [25]. Therefore, we confirmed that TNF-α could be used for discriminating diagnosis of pleural effusions, from TB and malignant outcomes.

Another Th1 subset cytokine, IFN-γ, secreted by activated lymphocytes enhances the anti-myobacterial properties of macrophages and induces TNF. Both IFN-γ and TNF-α present near the local concentrations area in pleural fluid of TB, which are useful for the bacilli elimination and granuloma formation [27]. In addition, activated by TNF, macrophages produce large amount of nitric oxide (NO), with which, could eliminate *M. tuberculosis* efficiently [28]. Our study showed a significant increase of IFN-γ (114.97 ± 27.85 ng/L) in pleural fluid of TB than that of malignant cases (87.15 ± 18.77 ng/L). With a cut off of 103.65(ng/L), it yielded mild sensitivity (80.9%) and specificity (81.4%), although it is less powerful than TNF-α.

However, when compared with IFN-γ and TNF-α, the potential diagnostic value of IL-10 was poorly investigated. According to Olobo’s report [28], pleural IL-10 was accumulated in TB. However, in this study, we found that there was no statistical significance of pleural level of IL-10 in TB and malignant pleural effusions.

In addition, ROC curve analysis indicates the area under the curve (AUC) of IFN-γ, TNF-α and ADA_2_ were 0.831, 0.976 and 0.844 respectively. Therefore we confirmed that all of these three bio parameters are of great reliability for the diagnosis of tuberculous pleural effusion. With the greatest AUC (0.976), the sensitivity, specificity and accuracy of TNF-α are 83%, 97% and 90% respectively. ADA_2_ yielded comparable diagnostical values, AUC was 0.844, sensitivity was 76.6%, specificity was 97%, and accuracy was 86.7%. With the sensitivity of 80.9% and specificity of 81.4%, IFN-γ was of less clinical accuracy than TNF-α and ADA_2_. However, IL-10 was found not to be useful for the diagnosis.

Based on the fact above, IFN-γ, TNF-α, ADA and ADA_2_ would be the preferred choice to be used to discriminate if the patient is TPE or MPE. But in the clinical practices, to avoid any misdiagnose of diseases, the discriminating diagnosis is determined by taking many factors into account, rather than by testing any single method [18]. The combinations of two or more biomarkers are required to be positive for a diagnosis to be made, which increased the specificity at the expense of sensitivity. Although TNF-α or ADA2 were shown to be effective when used individually for differential diagnosing of TPE from MPE, the combinations of these two biomarkers should result in an improved sensitivity or specificity in reality. Our findings supported that the combination of TNF-α and ADA_2_ result in the optimal sensitivity of 78.9%, specificity of 97.7% and accuracy of 88.3%. Therefore, the combinations of TNF-α and ADA2 could effectively address the challenge of distinguishing tuberculous pleural effusion from malignant pleural effusion.

## Conclusion

In conclusion, our investigation suggested that, compared to malignant pleural effusion, IFN-γ, TNF-α, ADA and ADA_2_ all increased in tuberculous pleural effusion. In addition, combinations of TNF-α and ADA2 yielded the optimal clinical accuracy on making differential diagnose between TPE and MPE.

The number of patients in this study would not be sufficient to deduce a conclusion for a diagnostic accuracy study. A future research with a larger sample analysis will be done to confirm the conclusion drawn in this study.

## Abbreviations

TPE: Tuberculous pleural effusion; MPE: Malignant pleural effusion; TNF-α: Tumor necrosis factor-alpha; IFN-γ: Interferon-gamma; IL-10: Interlukine-10; ADA_2_: Adenosine deaminase 2; ADA: Adenosine deaminase; IAP: Immunosuppressive acidic protein; ROC curve: Receiver operating characteristic curve.

## Competing interests

The authors declare that they have no competing interests.

## Authors’ contributions

ML was responsible for all data of the study and modified the manuscript. HW, XX, XW, JW and JW performed the trial and collected all data. HW and XX wrote the draft. All authors read and approved the final manuscript.
